# Research progress of ECT2 and RhoA-related signaling pathways in gynecological tumors

**DOI:** 10.3389/fcell.2025.1602649

**Published:** 2025-06-27

**Authors:** Liying Sheng, Meili Liang, Yueli Wang, Zhimei Zhou, Yajing Xie, Yumin Ke, Zhuna Wu

**Affiliations:** Department of Gynecology and Obstetrics, The Second Affiliated Hospital of Fujian Medical University, Quanzhou, China

**Keywords:** Ect2, RhoA, ROCK, ovarian cancer, cervical cancer, endometrial cancer

## Abstract

Epithelial Cell Transformation Factor 2 (ECT2) is highly expressed in a variety of cancers, including gynecological tumors. The mislocalization of ECT2 can abnormally activate Ras homolog family member A (RhoA) in the Ras homolog gene family (Rho) Guanine nucleotide Exchange Factor (GEF) family. Activated RhoA binds to Rho-associated protein kinase (ROCK), phosphorylates various target proteins, triggers a cascade reaction, regulates the functions of downstream proteins, and thereby plays an important role in the occurrence and development of tumors. This article reviews the roles of ECT2 and RhoA/ROCK signaling pathways in ovarian cancer, cervical cancer, and endometrial cancer, and summarizes and discusses the research progress of downstream molecules, transduction pathways, and mechanisms related to them. Through comprehensive analysis and summary of the current research results, it is revealed that the ECT2/RhoA/ROCK signaling pathway and related crosstalk pathways play an important role in the occurrence, development, and metastasis of gynecological tumors. This article aims to provide a basis for related research and offer relevant references for the treatment of gynecological tumors in the future.

## 1 Introduction

ECT2 is closely related to cell proliferation-related pathways. As a guanine prenucleotide change factor of Rho GTPase, high ECT2 is a factor for poor prognosis in cancer patients (Cook et al., 2014). ECT2 is overexpressed in various cancers such as non-small lung cell carcinoma, glioblastoma, and prostate cancer (Sano et al., 2006; Hirata et al., 2009; Guo et al., 2017). Generally speaking, a high ECT2 level is an unfavorable factor for the prognosis of patients. Recent studies have shown that ECT2 can abnormally activate RhoA, and then the activated RhoA binds to ROCK, participating in the abnormal proliferation and migration of various tumor cells by mediating various cascade reactions (Zubor et al., 2020; Saito et al., 2004; Huff et al., 2013). Furthermore, multiple crosstalk pathways interact between ECT and the RhoA/ROCK signaling pathway, jointly participating in the occurrence and development of tumors. However, few studies have explored the role of the ECT2 and RhoA/ROCK signaling pathways in gynecological malignancies. This article aims to summarize the research status and related mechanisms of ECT2 and RhoA/ROCK in gynecological tumor tissues and to analyze and explore the interactions of related crosstalk signal molecules and signal pathways in the growth, invasion, metastasis, and diffusion of gynecological malignant tumor cells. Based on these mechanisms, future research can combine clinicopathological data to identify and validate new markers in gynecological cancers. This may improve the early detection of gynecological malignancies and provide potential value for future research on therapeutic targets.

## 2 ECT2 and RhoA/ROCK signaling pathways

### 2.1 Structure and role of ECT2

**FIGURE 1:**
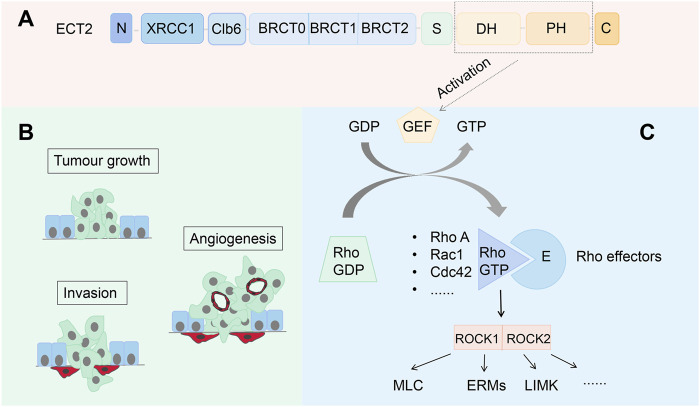
**(A)** Model of ECT2 structure; **(B)** Rho GTPase is involved in cell proliferation, invasion, and induction of tumor angiogenesis; **(C)** ECT2 catalyzes the conversion of GDP to GTP, which then binds to and activates the Rho protein; When RhoA binds to ROCK, it binds to or phosphorylates various target proteins to regulate the function of downstream proteins.

ECT2 is a GEF that plays a crucial role in cytokinesis (Tsuji et al., 2012). ECT2 contains multiple domains, the N-terminal domain is related to cell cycle regulators and is involved in cell cycle regulation. The N-terminal domain includes three tandem breast oncogene-1 carboxy terminus (BRCTs), namely, BRCT0, BRCT1, and BRCT2 (Sheng et al., 2011). Previous studies have suggested that the BRCT domain may be related to DNA damage response, cell cycle checkpoints, and DNA transcription. Therefore, if the structural changes in the BRCT domain and then cause ECT2 function mutations, it may lead to tumors or cancer (Huyton et al., 2000). Its N-terminal sequence also includes the X-ray repair cross complementing 1 (XRCC1) domain, a protein associated with repairing defective DNA strands (Thompson et al., 1990). Between the XRCC1 and BRCT domains is the Cyclin B6(Clb6) domain. In eukaryotic cells, the S phase is responsible for DNA damage repair and DNA replication. The occurrence of this cell cycle depends on cyclin-dependent kinase (CDK), and Clb6 is an important molecule that activates CDK (Palou et al., 2010). The other end of ECT2 is the C-terminal domain. There is a small domain connection between the N-terminal and C-terminal domains, also known as the S-loop, which contains a nuclear localization sequence (NLS) that regulates the intracellular localization of ECT2 (Solski et al., 2004). The C-terminus consists of Dbl homology (DH) and pleckstrin homology (PH) domains is the catalytic center of ECT2 and can activate RhoA GTPase (Solski et al., 2004) (Figure 1A). In the M phase, ECT2 is recruited to the central spindle and activates the Rho signaling pathway, which subsequently induces the formation and contraction of contractile rings (Niiya et al., 2006). Deletion of ECT2 interferes with cytokinesis but has no significant effect on mitosis. In addition to being involved in normal cell activity, ECT2 is also involved in cell malignant transformation, tumorigenesis, and metastasis (Saito et al., 2004). ECT2 contains the nuclear localization signal (NLS) and is mainly distributed in the nucleus (Justilien and Fields, 2009), it is independent of cytokinesis regulation, and nuclear ECT2 promotes cellular malignant transformation in other ways (Chen et al., 2015). In addition, ECT2 is a multifunctional oncogenic protein that upregulates Rho-extracellular signal-regulated kinase (Erk) signaling, promotes cell proliferation, inhibits apoptosis, and induces distant cell metastasis (Hall, 2012).

### 2.2 The function of Rho protein

Rho GTPases are molecular switches that act as signaling nodes to integrate extracellular signals and propagate intracellular signals. They control many normal cellular processes, including actomyosin remodeling, cell polarity, gene expression, and cell cycle progression (Ellenbroek and Collard, 2007). The most prominent members of this family are RhoA, ras-related C3 botulinum toxin substrate 1(Rac1), and cell division cycle 42(Cdc42). These Rho GTPases are often overexpressed or dysregulated in tumors (Ellenbroek and Collard, 2007). Rho GTPases regulate the cytoskeleton involved in the regulation of actin by regulating downstream effector protein activity (Aspenström et al., 2004; Ridley, 2006), thereby mediating vesicle trafficking to polarize cells (Halbleib and Nelson, 2006)and causing them to undergo a series of events such as cell shape change, cell adhesion, cell proliferation, and cell migration (Yoshioka et al., 1999). In addition, RhoA molecules localized to the cytoplasmic membrane are activated to signal ROCK and actomyosin to promote cell invasion (van Golen et al., 2002).In addition to this, Rho GTPase can also increase the production of angiogenesis, especially vascular endothelial growth factor (VEGF), which induces tumor angiogenesis and further promotes tumor cell metastasis (Saito et al., 2004). In conclusion, current studies have shown that Rho GTPase is involved in various processes of tumorigenesis and development in a variety of ways through multiple pathways (Figure 1B).

### 2.3 ECT2 can activate the RhoA/ROCK signaling pathway

The mislocalization of ECT2 can anomalously activate RhoA, Rac1, and Cdc42 in the Rho GTP family (Saito et al., 2004). GEF catalyzes the conversion of GDP to GTP and binds to Rho proteins. GDP-bound Rho proteins are in an off state, while GTP-bound proteins are in an open state, where they bind to their effector targets to exert effects and transmit downstream signals (Julian and Olson, 2014). Activated RhoA binds to ROCK, which then phosphorylates various target proteins or binds to certain target proteins to regulate the function and role of downstream proteins, which in turn leads to cell proliferation and migration (Huff et al., 2013) (Figure 1C). The NLS in the ECT2 domain locate it in the cell nucleus. At this stage, the N-terminal domain in ECT2 interacts with the catalytic domain to inhibit its exchange activity and isolate it from Rho GTPases. However, in tumor cells, the NLS structure of ECT2 is missing, resulting in its mislocalization into the cytoplasm, the disappearance of its auto-inhibitory effect, and then the activation of Rho family GTPase to drive transformation (Saito et al., 2004)**.**

## 3 Molecules and signaling pathways involved in ovarian cancer

The mortality rate of ovarian cancer ranks first among gynecological malignant tumors, and its poor prognosis is due to the insidious onset of the disease, no obvious specific symptoms in the early stage, and no specific screening method has been found at present, and about 70% of patients are already at an advanced stage when they are found. Therefore, a deeper understanding of the molecular process of early ovarian cancer and the identification of specific biomarkers can help predict and diagnose its occurrence earlier.

**FIGURE 2:**
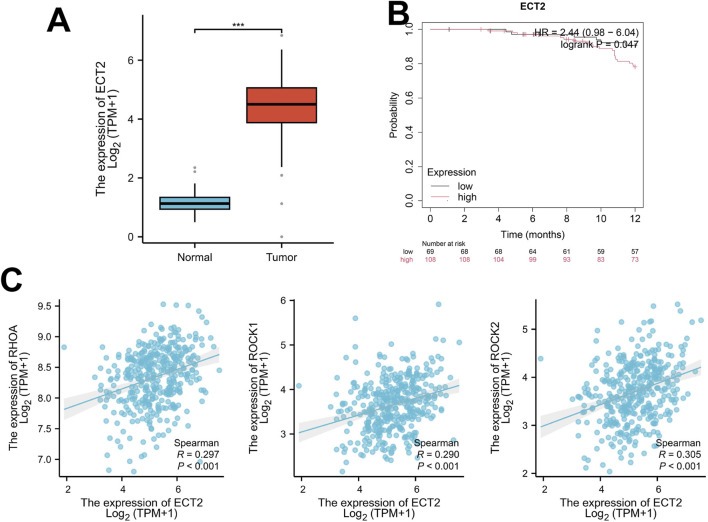
**(A)** The expression of ECT2 in ovarian cancer tissues is significantly higher than that in normal ovarian tissues; **(B)** The survival time of the ECT2 high-expression group was shorter than that of the ECT2 low-expression group; **(C)** The expression of ECT2 in ovarian cancer was significantly positively correlated with RhoA, ROCK1, and ROCK2.

By retrieving and obtaining transcriptome sequencing data from 427 ovarian cancer samples in the TCGA database (https://cancergenome.nih.gov/), significant differences were found in the expression level of ECT2 between ovarian cancer and normal ovary (Figure 2A). Survival analysis revealed that the expression level of ECT2 was associated with patient prognosis, and the survival time of patients in the high ECT2 expression group was shorter than that in the low ECT2 expression group (Figure 2B). And through the correlation analysis of the expressions of ECT2 and RHOA, ROCK1, and ROCK2 in ovarian cancer samples in TCGA database, it was found that the expression of ECT2 was significantly positively correlated with RhoA, ROCK1, and ROCK2 (Figure 2C).

### 3.1 ECT2 is highly expressed in ovarian cancer

Ovarian cancer is a malignancy with the second-highest frequency of ECT2 amplification, and ECT2 is also overexpressed at the mRNA level (Haverty et al., 2009). Bioinformatics analysis showed that ECT2, as a part of the 3q26 amplicon, co-amplified and overexpressed with (protein kinase C iota) PRKCI, and other molecules in ovarian serous cancer (Callebaut and Mornon, 1997; Wang et al., 2013). Genetic mutations in BRCA1 are known to predispose to breast and ovarian cancer (Mota et al., 2023), as an important tumor suppressor gene, BRCA1 plays a key role in the occurrence and development of breast cancer and ovarian cancer, and BRCA1 protein is composed of multiple domains, among which the BRCT domain is its core component (Ismail et al., 2024). Functionally, BRCA1 maintains genome integrity through homology-directed repair (HDR) and stall fork protection (SFP), while the recognition of phosphoric acid by the BRCT domain is a key link in the HDR and SFP pathways. Once there is a mutation in the BRCT domain, HDR and SFP will fail at the same time, resulting in impaired mechanisms for maintaining genomic stability (Zubor et al., 2020). As mentioned above, the ECT2 domain includes three tandem BRCTs, which may be one of the important mechanisms by which ECT2 is involved in the occurrence and development of ovarian cancer. An experimental result showed that ECT2 can act as GEF to mediate the transformation and growth of tumor cells by activating Rac in the nucleus and RhoA in the cytoplasm; In cells, nuclear-localized ECT2 and its Rho GEF activity can promote the growth and transformation of ovarian cancer cells; However, cytoplasmically localized ECT2 does not promote the transformation of cancer cells, but may counteract its role in the nucleus (Haverty et al., 2009).

### 3.2 The occurrence and progression of ovarian cancer are related to the RhoA/ROCK signaling pathway

In one study, the expression level of RhoA mRNA was quantified using real-time polymerase chain reaction (RT-PCR) and Western blotting (WB). It found a strong response band of RhoA mRNA was observed after reverse transcription(RT) response from ovarian malignant tumors, while weak response bands were observed in benign or borderline tumors, indicating that the expression level of RhoA mRNA in ovarian malignant tumors was significantly higher than benign and borderline tumors (Horiuchi et al., 2003; Zaoui et al., 2019). In the histological subtypes of ovarian cancer, the expression level of RhoA mRNA in serous carcinoma is significantly higher than that of endometrial or clear cell carcinoma (Barrès et al., 2010). One case study found that in the FIGO stages of ovarian cancer (Shepherd, 1989), the expression levels of RhoA mRNA were significantly higher in stage III-IV tumors than in stage I-II (Ellenbroek and Collard, 2007). In addition, the response band of ROCK was observed in all ovarian tumors, but there was no significant difference in benign, borderline, or malignant tumors (Barrès et al., 2010).

**FIGURE 3:**
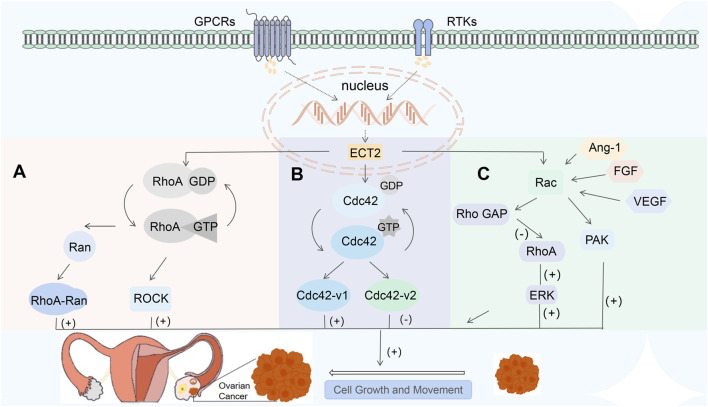
**(A)** RhoA is involved in cell metastasis by forming complexes with RAN and activating ROCK to regulate the mechanosensitivity of tumor cells; **(B)** ECT2 can bind Cdc42 and GTP to activate various downstream effectors; Cdc42-v1 and Cdc42-v2 have the opposite effect; **(C)** Rac is activated by VEGF, Ang-1, FGF, *etc.*, which then mediates cascades of downstream molecules; Rac and RhoA are antagonistic to each other.

RhoA GTPase can form a complex with the small GTPase nucleocytoplasmic shuttle protein Ras-associated nucleoprotein (Ran), which is closely related to the occurrence, progression, survival, and recurrence of epithelial ovarian cancer (EOC) (Horiuchi et al., 2003). The comparative analysis of primary and metastatic lesions of ovarian cancer showed that the expression level of RhoA mRNA in metastatic lesions was significantly higher than that of primary lesions (Barrès et al., 2010). Ran regulates cell proliferation and migration or invasion through RhoA recruitment (Horiuchi et al., 2003), This may be the mechanism for its higher expression in metastatic lesions. It has been reported that the depletion of Ran can reduce tumor cell migration, prevent EOC cell proliferation, and inhibit their growth (McGrail et al., 2014; Liu et al., 2023). These suggest that the Ran-RhoA signaling complex may be a molecular target to control ovarian cancer metastasis and may be a potential therapeutic pathway. Previous studies have shown that ovarian cancer cells are more likely to metastasize through peritoneal implantation, preferentially proliferating and metastasizing in soft tissues, such as fat cells, which have an abundant source of energy. Further studies have shown that this phenomenon may be due to its chemical and mechanical sensitivities (Sharma et al., 2012). The mechanistic sensitivity is related to the RhoA-ROCK signaling pathway (Horiuchi et al., 2003; Sharma et al., 2012). Fat cells secrete more leptin, which functions through the leptin receptor (Ob-Rb) (Ghasemi et al., 2017). After leptin binds to OB-Rb on the surface of ovarian cancer cells, it can increase the expression of RhoA and activate the RhoA-ROCK pathway. On the other hand, studies have found that soft matrices can alter the local mechanical properties of the cellular environment and the density of binding sites, thereby optimizing cell adhesion in local areas and further enhancing the generation of intracellular internal forces (Sun et al., 2022; Maheshwari et al., 2000). Enhanced intracellular internal forces can activate the RhoA-ROCK pathway (Sun et al., 2022). These two pathways activate the RhoA-ROCK pathway and induce the activation of myosin phosphatase targeting subunit 1(MYPT1) and urokinase (uPA) (Ghasemi et al., 2017; Leve et al., 2011; Kato et al., 2015). MMP7 and uPA can reorganize the cytoskeleton, stimulate the formation of stress fibers and focal adhesion complexes, and regulate cell movement to affect the invasion and metastasis of tumor cells (Kato et al., 2015). In this study, the occurrence of metastasis was reduced by inhibiting the RhoA-ROCK signaling pathway to reduce the mechanosensitivity of tumor cells (Sharma et al., 2012). And, cisplatin resistance in ovarian cancer has been reported to be related to its mechanistic nature (Solski et al., 2004). These studies have shown that the Rho-ROCK signaling pathway is a potential therapeutic pathway for ovarian cancer, and it is expected that new drugs will be developed as a target (Figure 3A).

### 3.3 Other crosstalk pathways mediated by ECT2

Sequences outside the DH and PH domains in ECT2 may affect the GTPase specificity of ECT2, causing it to activate not only RhoA but also Rac and Cdc42 *in vivo* (Cerione, 2004). CDC42 gene is involved in the regulation of various cellular processes such as cytoskeletal remodeling, cell migration, proliferation, and adhesion (Sinha and Yang, 2008). GEF converts Cdc42 from the inactive GDP-binding form to the active GTP-binding form. Conversely, the activated Cdc42 mediates the signal cascade reaction, leading to the activation of various downstream effectors and thereby participating in the tumorigenesis process (He et al., 2015). Previous studies have found that on the one hand, Cdc42 promotes tumor transformation, tumor invasion, and metastasis (Yang et al., 2007); on the other hand, the knockdown of the CDC42 gene leads to dysregulated expression of key transcription factors that promote tumorigenesis or partial transformation (Hudson et al., 2018). This suggests that the CDC42 gene is also a tumor suppressor. It is not clear what mechanism causes this contradiction. The human Cdc42 gene encodes two distinct Cdc42 variants, Cdc42-v1 and Cdc42-v2. Previous studies have hypothesized that: the tumor-promoting activity of the CDC42 gene is derived from CDC42-V1, while the tumor-suppressive activity is derived from CDC42-V2 (Yang et al., 2007), but there is a lack of research on its related mechanisms. (Figure 3B).

The small GTPase Ras-related C3 botulinum toxin substrate (Rac) is the intersection of multiple signaling pathways, which is involved in cell proliferation, apoptosis, differentiation, tumor metastasis, and regulates cell-environment interactions (Wang et al., 2018). Existing studies have confirmed that Rac is highly expressed in EOC (Leng et al., 2015). Excessive activation of Rac is an important driving factor for ovarian cancer. Inhibiting Rac activity or knocking down Rac expression can suppress the migration and invasion of ovarian cancer cells (Hudson et al., 2018). Previous studies have shown that nuclear Rac activity can rescue defects in ECT2-mediated transformation growth (Haverty et al., 2009). Angiogenesis promotes tumor growth by providing essential nutrients to tumor cells (Scott et al., 2010), and Rac is an essential part of the angiogenesis process (Zhu et al., 2004). Rac is activated by vascular endothelial growth factor (VEGF), angiogenin 1 (Ang-1), and fibroblast growth factor (FGF) (Bid et al., 2013), thereby regulating cell adhesion, cell proliferation and migration, and participating in tumor vascular invasion (Brantley-Sieders et al., 2009). Extracellular signaling mediated by various cell surface receptors, such as integrins, cytokine receptors, G protein-coupled receptors (GPCRs), cadherins, and receptor tyrosine kinases (RTKs), can enable ECT2 to function to recruit Rac from the cytoplasm to the plasma membrane or other cellular locations (Chan et al., 2005; Bensen and Brognard, 2021); Rac then activates downstream effector molecules, including various proteins, kinases, and adaptor proteins that mediate subsequent cascade reactions, such as p21-activated kinase (PAK), *etc.* (Brantley-Sieders et al., 2009). The activity of Rho family GTPases is determined by three different types of regulatory molecules: specific GEF, GTPase-activating proteins (GAP), and guanine nucleotide dissociation inhibitors (GDI). GEF activates Rho GTPase by catalyzing the exchange between the bound GDP molecules and GTP, while GAP stimulates the intrinsic GTPase activity of Rho proteins, thereby restoring them to an inactive GDP-binding state (Bos et al., 2007). GDI is responsible for maintaining a stable and inactive cytoplasmic bank of Rho GTP enzymes (Garcia-Mata et al., 2011). Rac GAP can inactivate Rac by reducing the level of GPT-RAC. Interestingly, the level of RhoA was also upregulated in EOC cells overexpressing Rac GAP; Similarly, Rac GAP is positively correlated with the activity and expression of the downstream effector Erk protein of RhoA. Conversely, knocking down the Rac GAP inhibited the level of RhoA (Wang et al., 2018). As the activity of Rac gradually increases, the Rho GAP mediated by Rac will be activated, and then the activity of RhoA will decrease (Lawson and Burridge, 2014). This indicates that there is a negative crosstalk between Rac and RhoA. Current researchers are still unable to explain why Rac and RhoA antagonize each other but simultaneously promote the occurrence and development of ovarian cancer. The mechanism therein awaits further study (Figure 3C).

## 4 Molecules and signaling pathways involved in cervical cancer and human papillomavirus (HPV)

Cervical cancer is still the fourth most common cancer in women worldwide, and the occurrence of cervical cancer is associated with persistent HPV virus infection. Therefore, it is particularly important to elucidate the mechanism of the occurrence and progression of cervical cancer and explore new treatments.

**FIGURE 4:**
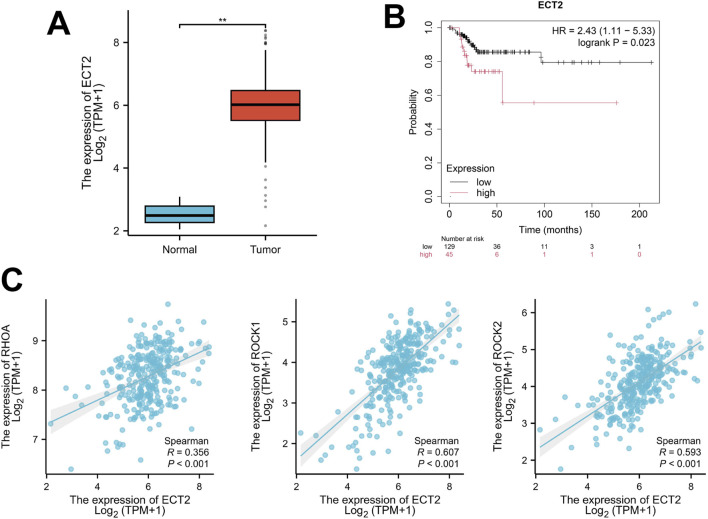
**(A)** The expression of ECT2 in cervical cancer tissues is significantly higher than that in normal cervical tissues; **(B)** The survival time of the ECT2 high-expression group was shorter than that of the ECT2 low-expression group; **(C)** The expression of ECT2 in cervical cancer was significantly positively correlated with RhoA, ROCK1, and ROCK2.

By retrieving and obtaining transcriptome sequencing data from 306 cervical cancer samples in the TCGA database (https://cancergenome.nih.gov/), significant differences were found in the expression level of ECT2 between cervical cancer and normal cervix (Figure 4A). Moreover, survival analysis revealed that the expression level of ECT2 was related to the prognosis of patients. The survival time of patients in the high expression group of ECT2 was shorter than that in the low expression group of ECT2 (Figure 4B). And through the correlation analysis of the expressions of ECT2 and RHOA, ROCK1, and ROCK2 in cervical cancer samples in TCGA database, it was found that the expression of ECT2 was significantly positively correlated with RhoA, ROCK1, and ROCK2 (Figure 4C).

### 4.1 ECT2 is highly expressed in cervical cancer

Squamous cell carcinoma of the cervix accounts for 75%–80% of cervical cancers. The effect of ECT2 on cervical cancer is currently unknown. Previous studies have shown that chromosome 3q contains many oncogenes that determine squamous cell differentiation, including ECT2, and its amplification is the most common chromosomal aberration in most squamous cell carcinomas, and it is also a key gene that leads to squamous cell differentiation (Hudson et al., 2018; Sun et al., 2020). ECT2 regulates apoptosis after DNA damage in cervical cell lines (Chen et al., 2022), and the expression of this gene increases with the onset of DNA synthesis (Chen et al., 2015). ECT2 is the gene with the highest -fold upregulation in chromosome 3q, making it a good candidate for a driver oncogene in cervical cancer cells (Chen et al., 2022). Previous studies downloaded gene expression data from Gene Expression Omnibus (GEO) normal cervical tissues and cervical cancer tissues and applied bioinformatics analysis to show that the expression level of ECT2 mRNA in cervical cancer tissues was higher than that in normal cervical tissues based on GSE9750, GSE29570, and GSE52903 datasets (Sharma et al., 2012). Some studies have found that after the knockdown of ECT2, the proliferation rate of cervical cancer cells decreases; However, after overexpression of ECT2, the proliferation rate of cervical cancer cells increases (Amaya et al., 2017). In addition, one study significantly improved the migration and invasion ability of ECT2 after increasing its expression. After injecting cancer cells with high ECT2 expression into mice, the volume and weight of tumors in mice increased significantly compared to the control group (Sharma et al., 2012). These results suggest that ECT2 may act as a pro-tumor gene to promote cervical cancer cell proliferation.

### 4.2 ECT promotes the proliferation and migration of cervical cancer cells through the RhoA pathway

**FIGURE 5:**
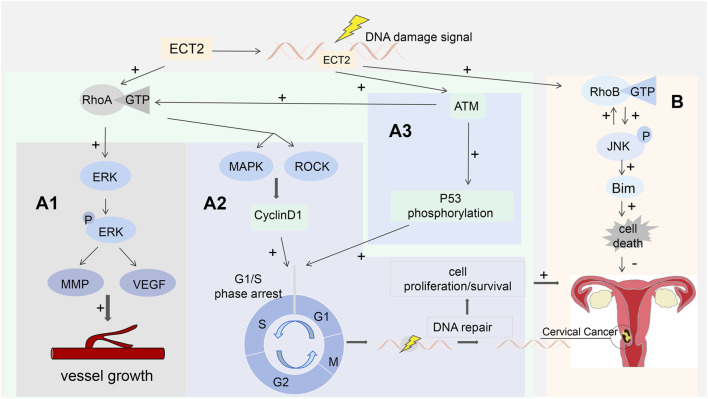
**(A1)** ECT2 binds RhoA to GTP, which then activates ERK to produce p-ERK, which in turn increases the expression levels of MMP and VEGF; **(A2)** ATM activates RhoA and mediates the MAPK and ROCK pathways to regulate the expression level of cyclin D1 and participate in the DDR process; **(A3)** p53 is the substrate of ATM participating in DDR; ECT2 assists in ATM-mediated phosphorylation of p53; **(B)** The ECT2-RhoB-JNK pathway leads to cell death by triggering the pro-apoptotic protein Bim.

Previous reports have shown that ECT2 has Rho GTPase activity, which may promote the occurrence and progression of squamous cell carcinoma by mediating the overexpression of Rho A (Mendez and Ramirez, 2013; Chen et al., 2015; Sun et al., 2020). Previous studies have shown that the expression of RhoA, ROCK-1, and ROCK-2 is significantly elevated in cervical cancer cells. Also, the expression of RhoA was higher in patient specimens of vascular invasion or lymph node metastasis. In this study, the methods of functional gain and loss of function were used to further verify the promoting effect of RhoA in the proliferation of cervical cancer cells: The proliferation of Hela cells overexpressing RhoA in the experimental group was significantly higher than that in the control group. RhoA overexpression in Hela (RhoA+) cells was then blocked with RhoA-specific siRNA, and the proliferation of Hela (RhoA+) cells was significantly lower than that of the control group, and it was determined that siRNA transfection reversed the promotion of HeLa cell proliferation by overexpressing RhoA (Mendez and Ramirez, 2013). Previous studies have confirmed that ECT2 promotes the development and metastasis of hepatocellular carcinoma and esophageal squamous cells by modulating the RhoA/ERK signaling axis (Xu et al., 2013). ECT2 may be also involved in the occurrence and progression of cervical cancer through this signaling pathway, but no further studies have confirmed this so far. Rho GTPases are thought to be able to modulate the release of pro-angiogenic factors to promote neovascularization (Sun et al., 2020), and induction of tumor vascularization plays a key role in promoting the progression and metastasis of malignant tumors. Matrix metalloproteinases (MMPs) and VEGF are involved in angiogenesis, metastasis, and tumor cell proliferation (Tanaka et al., 2020). Overexpression of ECT2 can activate RhoA binding to GTP, which can then activate ERK to produce p-ERK (Sun et al., 2020; Cao et al., 2021). Activation of p-ERK can greatly increase the expression levels of MMP and VEGF in cervical cancer cell lines (Tanaka et al., 2020). This suggests that inducing tumor vascularization may act through a signaling pathway composed of ECT2, RhoA, RhoA-GTP, ERK, and VEGF/MMP. (Figure 5A1).

In terms of treatment, previous studies have shown that ECT2 in cancer cells overcomes endogenous DNA damage by promoting double-strand break (DSB) repair to maintain their survival and reduce the effect of radiotherapy or chemotherapy (Liu et al., 2014). In patients with FIGO stage III, RhoA overexpression was associated with significantly worse disease-free survival (DFS) and long-distance metastasis-free survival (DMFS); It is also associated with distant metastases after concurrent chemoradiotherapy (CCRT); These may be related to the ECT-RhoA-ROCK signaling pathway (Liu et al., 2014; Osaki et al., 2016; Sun et al., 2020). Ionizing radiation can inhibit cell metastasis in which RhoA activity decreases, and RhoA overactivation causes cells to metastasize after radiation therapy (Ridley, 2001). This suggests that the regulation of RhoA activity affects the sensitivity of cervical cancer cells to radiation therapy (Mordes et al., 2008). ECT2 and RhoA can be used as potential therapeutic targets for the reversal of tolerance to radiotherapy and chemotherapy for cervical cancer. The N-terminal BRCT domain of ECT2 is highly similar to the sequence of DNA topoisomerase 2-binding protein 1 (Sheng et al., 2011). Topoisomerase 2-binding protein 1 is an important regulator of DNA damage response (DDR) (Shiloh, 2001). As a result, ECT2 recruits damaged chromatin to participate in DSB repair and DDR processes. Ataxia - Telangiectasia Mutated (ATM) kinases are key proteins responsible for detecting DNA damage and activating cellular responses to avoid genetic instability (Cheng et al., 2021). ATM-dependent activation of RhoA is involved in the DDR process by regulating the expression level of cyclin D1 through its downstream mitogen-activated protein kinase (MAPK) and ROCK (Paull, 2015) (Figure 5A2). p53 is a substrate in which ATM participates in DDR (He et al., 2016). ECT2 acts as an adaptor protein to assist ATM-mediated phosphorylation of p53 (Srougi and Burridge, 2011) (Figure 5A3).

### 4.3 ECT mediates other crosstalk pathways and cervical cancer

Preliminary studies have shown that DNA damage-mediated activation of ATM can trigger ECT2 localization to the cytoplasm, and ECT2 in the cytoplasm can activate RhoB (Chen et al., 2000). Many studies have shown that RhoB may have antitumor activity and can inhibit the malignant transformation of tumor cells (Mazieres et al., 2004). Re-expression of RhoB reduces proliferation and tumor growth *in vivo* (Xie et al., 2019). Cyclin B1 plays a key role in inhibiting apoptosis in tumor cells (Kamasani et al., 2004). RhoB can inhibit the activity of the cyclin B1 promoter to reduce its expression level, which in turn leads to cervical cancer cell death (Sun et al., 2002). C-Jun N-terminal kinase (JNK) is a downstream signal of RhoB, which can directly upregulate the expression level of RhoB and/or increase the levels of GEF and ECT2 to increase the activity of RhoB, thereby phosphorylating JNK, triggering the pro-apoptotic protein Bim and leading to cell death; it has been speculated that there is a positive feedback relationship between them, with RhoB activity stimulating sustained JNK phosphorylation, thereby enhancing cervical cancer cell death through upregulation of RhoB (Chen et al., 2000) (Figure 5B). It has been proven that ECT2 also promotes cervical cancer progression by regulating the AKT/mTOR pathway, and the expression levels of p-AKT and p-mTOR are also significantly increased in cells overexpressing ECT2, while the opposite is true for underexpressing ECT2. ECT2 can directly bind to AKT to form the ECT2/AKT complex, which in turn promotes the phosphorylation of AKT, thereby regulating the downstream mTOR pathway. To further validate the relationship between them, the accelerated proliferation and reduced apoptosis induced by ECT2 overexpression were negated by the transfection of si-AKT into ECT2-overexpressing cervical cancer cells; These results suggest that the downregulation of AKT can reverse the anti-apoptosis, proliferation and activation of the AKT/mTOR pathway induced by ECT2 overexpression, and further reveal that ECT2 promotes the malignant progression of cervical cancer by regulating the AKT/mTOR pathway (Sharma et al., 2012). ECT2 can mediate a variety of signaling pathways involved in the progression of cervical cancer, and other pathways are still under investigation.

### 4.4 RhoA is associated with high-risk HPV infection

**FIGURE 6:**
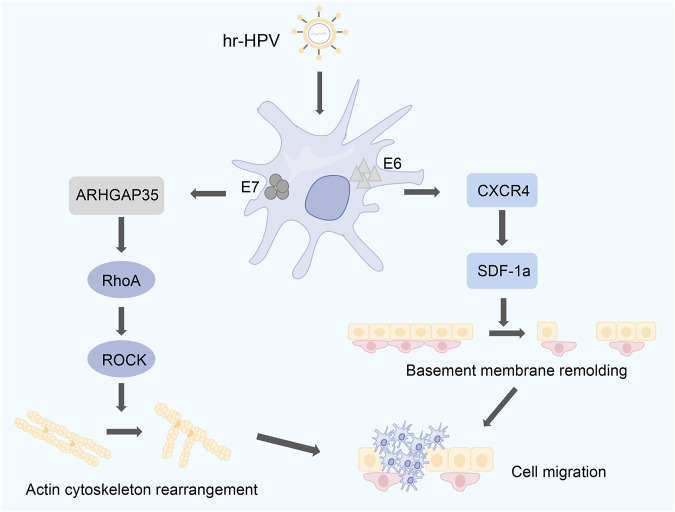
The carcinogenic potential of hr-HPV is associated with E6 and E7. E6 is associated with high expression of RhoA. E7 regulates the expression levels of RhoA and Rac1 through ARHGAP35. SDF-1α stimulates CXCR4, which in turn activates the RhoA/ROCK-mediated cascade. On the one hand, the interaction between SDF-1α and CXCR4 can remodel the basement membrane of cells. On the other hand, the RhoA/ROCK-mediated cascade can reconstitute the actin cytoskeleton.

Previous reports have shown no correlation between RhoA expression and age, sex, tumor size, and HPV infection, but a positive correlation with FIGO stage, vascular invasion, and lymphatic metastasis (Mendez and Ramirez, 2013; Osaki et al., 2016). In another report, RhoGTPase activity was increased in SiHa, a high-risk human papillomavirus (hr-HPV)-infected cervical cell line. The oncogenic potential of hr-HPV is attributed to its E6 and E7 tumor proteins, and the presence of E6 tumor proteins is associated with increased RhoGTPase activity and RhoA expression. RhoA GTPase is negatively regulated by ARHGAP35 (p190GAP family) and is ARHGAP35 one of the targets of E7, and HPV E7 is involved in cell migration by regulating RhoA and Rac1 (Zaoui et al., 2019). High expression of some chemokines such as SDF-1α and its migration-promoting effect on HeLa cells have been identified early in cervical cancer cells (Zhang et al., 2007). C-X-C chemokine receptor type 4 (CXCR4) was also found to be highly expressed in HPV-positive cervical cancer cells (Bleul et al., 1996). CXCR4 is a chemokine receptor, whereas stromal cell-derived factor-1α (SDF-1α) is the only chemokine of CXCR4 (Peng et al., 2005). The binding of SDF-1α to CXCR4 results in calcium mobilization, reduction of intracellular cyclic AMP/AMP proteins, and activation of multiple signal transduction pathways (Bartolomé et al., 2004). Among them, SDF-1α incubation stimulates CXCR4, which in turn activates the RhoA/ROCK-mediated cascade in cancer cells (Kaibuchi et al., 1999; Amine et al., 2009). On the one hand, the interaction between SDF-1α and CXCR4 can remodel the basement membrane and degrade the basement membrane (Amine et al., 2009); On the other hand, in harmony with the degradation of the basement membrane, the RhoA/ROCK-mediated cascade can reorganize the actin cytoskeleton and activate the motility mechanism of tumor cells (Baiden-Amissah et al., 2021). The combination of these two aspects may be the mechanism that leads to the trans-basement membrane invasion of HPV-positive cervical cancer cells (Figure 6). The mechanism by which Rho GTPase signaling interacts with human papillomavirus proteins is still under investigation.

## 5 Role and mechanism of RhoA/ROCK in endometrial cancer

The overall 5-year survival rate for endometrial cancer (EC) in the early stages ranges from 74% to 91%, but the survival rate for advanced-stage cancer is only 20%–26% because there is no effective treatment in advanced and metastatic cases. ECs are divided into two groups by histopathological features, type I includes estrogen-dependent tumors, type II is hormone-independent tumors, and type I is more common, accounting for 80% of all tumors (Hao et al., 2015). Distant metastases are the most common cause of death from endometrial cancer (Izdebska et al., 2018). Therefore, it is important to study the biomarkers and molecular mechanisms related to endometrial cancer cell migration.

**FIGURE 7:**
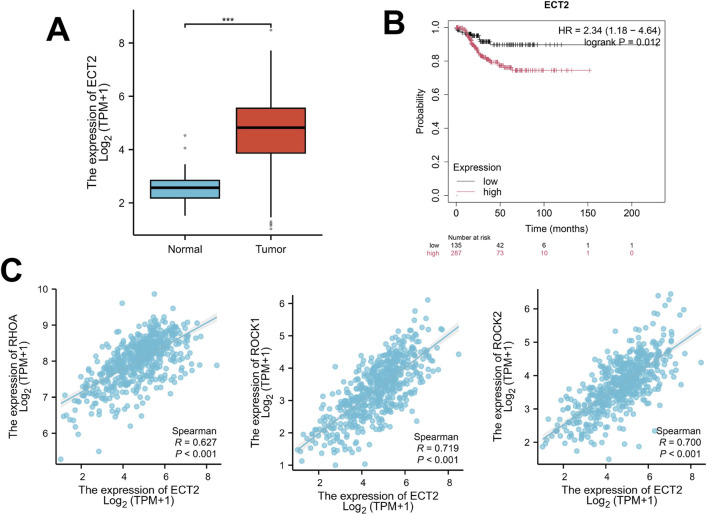
**(A)** The expression of ECT2 in endometrial cancer tissues is significantly higher than that in normal endometrial tissues; **(B)** The survival time of the ECT2 high-expression group was shorter than that of the ECT2 low-expression group; **(C)** The expression of ECT2 in endometrial cancer was significantly positively correlated with RhoA, ROCK1, and ROCK2.

By retrieving and obtaining transcriptome sequencing data from 554 endometrial cancer samples in the TCGA database (https://cancergenome.nih.gov/), significant differences were found in the expression level of ECT2 between endometrial cancer and normal endometrium (Figure 7A), Moreover, survival analysis revealed that the expression level of ECT2 was related to the prognosis of patients. The survival time of patients in the high expression group of ECT2 was shorter than that in the low expression group of ECT2 (Figure 7B). And through the correlation analysis of the expressions of ECT2 and RhoA, ROCK1, and ROCK2 in endometrial cancer samples in TCGA database, it was found that the expression of ECT2 was significantly positively correlated with RHOA, ROCK1, and ROCK2 (Figure 7C).

### 5.1 The expression of ECT2 is elevated in endometrial cancer

A study found through the analysis of differentially expressed gene (DEGs) data that ECT2 was highly expressed in atypical endometrial hyperplasia (AH) and endometrial cancer. ECT2 has high accuracy in differentiating benign and malignant endometrium and is expected to become a promising biomarker for the diagnosis of endometrial cancer and a prognostic indicator for endometrial cancer (Wu et al., 2024).

### 5.2 RhoA/ROCK is involved in endometrial cancer by mediating calcium concentrations

Previous studies have shown that aberrant activity of actin cytoskeletal regulatory proteins may have an impact on cell adhesion and migration, leading to increased cancer cell mobility, promoting accelerated disease progression, and subsequently accelerated metastasis formation (Horiuchi et al., 2003; Yamazaki et al., 2005; Fiorio Pla and Gkika, 2013). The calcium channel Transient Receptor Potential Vanilloid 4 (TRPV4) is a member of the transient receptor potential (TRP) family that regulates calcium homeostasis in living organisms (Stewart et al., 2015). Calcium ions play an important role in the changes in the actin cytoskeleton. It has been reported that the concentration of cytoplasmic free calcium ions regulates cancer cell function (Li et al., 2019). The increase in calcium ionization promotes the metastasis of abdominal tumor cells and the metastasis of lymph nodes in endometrial cancer (Schwartz and Skinner, 2012; Li et al., 2020b). In estrogen-dependent endometrial cancer, estrogen may work synergistically with calcium (Mo and Yang, 2018). Stimulated by estrogen, calcium ions flow through L-type calcium channels (LTCC) and promote the proliferation and migration of endometrial cancer cells (Hao et al., 2015). Overactive calcium channels may promote cancer cell metastasis and colonization by reorganizing the actin cytoskeleton, degrading the extracellular matrix, and remodeling the tumor microenvironment (Li et al., 2020a). The levels of free calcium and TRPV4 in the cancer cells of endometrial cancer FIGO stage II-IV were significantly higher than those in stage I (Déliot and Constantin, 2015). In most cases, increased expression and/or activity of calcium channels maintain high levels of calcium ions, promoting cancer cell proliferation and migration (Fife et al., 2014). To confirm the role of TRPV4-mediated calcium ions in endometrial cancer, in a 2020 experiment, after inhibiting the expression of TRPV4 *in vitro*, cell motility decreased, and conversely, overexpression of TRPV4 enhanced the motility of endometrial cancer cells; in this study, EC cells with high expression of TRPV4 and antagonistic TRPV4 were constructed and injected into nude mice, and it was found that the number of metastatic peritoneal nodules in the high-expression group increased, while the number of metastatic peritoneal nodules in the antagonistic group decreased significantly (Déliot and Constantin, 2015). This study verified that TRPV4 is closely related to the occurrence and development of endometrial cancer both *in vitro* and *in vivo*.

**FIGURE 8:**
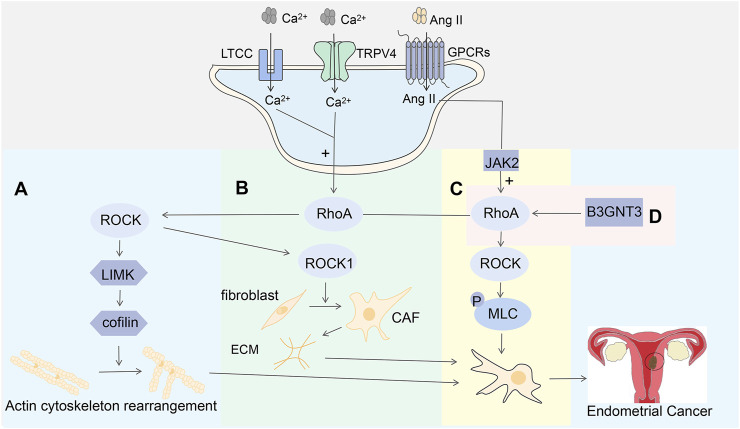
**(A)** Calcium influx from TRPV4 and L-type calcium channels activates the RhoA-ROCK-LIMK-cofilin pathway involved in cytoskeletal regulation; **(B)** The RhoA-ROCK1 signaling pathway promotes the transformation of fibroblasts to CAF, which in turn leads to physical remodeling of the pro-aggressive ECM; **(C)** Ang II enhances cell contraction by activating the JAK2-RhoA-ROCK-MLC signaling pathway through G protein-coupled receptors; **(D)** B3GNT3 is expressed at high levels in EC cells, where it mediates the regulation of the RhoA-RAC1 signaling pathway.

One of the most common mechanisms that regulate actin is through the Rho GTPase pathway, which may be triggered by calcium influx (Li and Wang, 2020). In endometrial cancer cells, the expression of RhoA and its downstream effector molecule ROCK was caused by a high concentration of free calcium ions, and its expression level increased with the increase of calcium ion concentration. However, the expression level of calcium ions decreased after the function of calcium ions was hindered by calcium chelators (Déliot and Constantin, 2015). RhoA-ROCK signaling is a key regulator of actomyosin contractility, which regulates cell shape, cytoskeletal arrangement, and thus cell functions such as cell proliferation, differentiation, motility, and adhesion (Maekawa et al., 1999). These studies suggest that the influx of TRPV4 and calcium ions activates the RhoA/ROCK pathway in EC cells, triggering the mechanism of cytoskeletal changes, which in turn mediates tumor cell migration. Western blotting showed that the expression levels of LIM domain kinase (LIMK) and cofilin were altered by the activity of TRPV4, and the levels of ROCK were also increased after ROCK overexpression. This suggests that LIMK and cofilin are key downstream components of ROCK in regulating the actin cytoskeleton. RhoA activation increases the expression of ROCK1 and further activates LIMK, which in turn phosphorylates cofilin, inhibits actin depolymerization activity, and stabilizes agonist stress fibers (Maekawa et al., 1999). These findings demonstrate that cytoskeletal regulation initiated by TRPV4 and calcium ion influx may act through the RhoA/ROCK/LIMK/cofilin signaling pathway (Figure 8A).

### 5.3 RhoA/ROCK is involved in endometrial cancer through other mechanisms

ROCK is one of the most important effector molecules downstream of RhoA, and it has two isoforms in cells: ROCK1 and ROCK2.ROCK1 is mainly involved in the formation of stress fibers, while ROCK2 is mainly involved in the process of cell phagocytosis and cell contraction (Wang et al., 2009; Bussard et al., 2016). Cancer-associated fibroblasts (CAFs) have a significant impact on cancer progression by remodeling extracellular matrix proteins (ECMs) and secreting various cytokines to stimulate cancer cell proliferation and cell migration (Subramaniam et al., 2013). Previous studies have demonstrated that CAF has pro-cancer activity (Bussard et al., 2016), it promotes the growth, migration, and invasion of EC tumors by secreting pro-tumor factors, including IL-6, IL-8, matrix-derived factor-1 α, VEGF, etc., (Orimo et al., 2001; Matsubara and Bissell, 2016). RhoA activation of ROCK1 is involved in the formation of stress fibers in CAF (Sanz-Moreno et al., 2011). RhoA/ROCK1 signaling-activated actin contractility promotes the conversion of fibroblasts to CAFs, which in turn leads to physical remodeling of the pro-invasive ECM, favoring tumor aggressiveness and dissemination (Mierke et al., 2008) (Figure 8B).

In addition, in smooth muscle and non-muscle cells, contraction is driven by the myosin II motor protein complex. When myosin II regulates myosin II regulatory light chain(MLC) phosphorylation, the myosin II motor protein complex is activated, which in turn activates myosin ATPase, thereby enhancing cell contractility (Fields and Justilien, 2010). Active ROCK can also affect cell migration by affecting MLC phosphorylation and activity (Yoneda et al., 2005).ROCK increases MLC phosphorylation, which contributes to actin recombination and stress fiber formation (Guilluy et al., 2010). Angiotensin II (Ang II) activates Janus kinase 2 (JAK2) through G protein-coupled receptors, which can phosphorylate Rho GTPase, which in turn activates RhoA, leading to the activation of ROCK and MLC phosphorylation (Zhang et al., 2015) (Figure 8C). Current studies have shown that β-1,3-N-acetylglucosamine transferase-3 (B3GNT3) is an oncogene that affects the energy metabolism of tumors by regulating galactosyltransferase, thereby affecting the survival and metastasis of tumor cells (Tang et al., 2019). High levels of B3GNT3 expression were monitored in endometrial cancer cells (Wang et al., 2021). In previous studies, it was observed that downregulation of B3GNT3 expression levels significantly reduced the expression levels of RhoA and RAC1 (Kim et al., 2005). These suggest that B3GNT3 may promote the migration and invasion of EC cells by modulating the RhoA/RAC1 signaling pathway (Figure 8D), but the upstream and downstream signaling molecules involved in this study remain to be studied.

## 6 Conclusion

This review comprehensively elucidates the intricate roles and contemporary research advancements of the ECT2 and RhoA/ROCK signaling pathways in the pathogenesis and progression of gynecological malignancies. ECT2, firmly established as an oncogene, exhibits consistent overexpression across ovarian, cervical, and endometrial cancers, a phenomenon intricately intertwined with tumor initiation, malignant progression, and adverse patient prognosis. Through the activation of the RhoA/ROCK signaling cascade, ECT2 orchestrates a series of oncogenic processes, including tumorigenesis, unrestrained proliferation, invasion, and metastasis. The RhoA/ROCK pathway, in turn, propels the development of gynecological tumors via a complex network of multi-tiered cascades and extensive molecular crosstalk. These signaling axes converge on pivotal aspects of tumor biology, encompassing angiogenesis, mechanical sensing, cytoskeletal remodeling, and DNA damage response, where they engage in dynamic synergistic or antagonistic interactions that govern cancer cell growth, dissemination, and colonization (Table 1).

**TABLE 1:** Summary of the expression of different molecules in gynaecological cancers.

Molecules	Expression levels in different types of gynecological cancers		
	Ovarian cancer	Cervical cancer	Endometrial cancer
ECT2	Overexpressed (Haverty et al., 2009)	High expression (Sun et al., 2020)	High expression (Wu et al., 2024)
RhoA	High expression (Horiuchi et al., 2003)	Overexpressed (Liu et al., 2014)	High expression (Zubor et al., 2020)
ROCK	General expression (Horiuchi et al., 2003)	Overexpressed (Liu et al., 2014)	High expression (Zubor et al., 2020)
Molecules in the relevant signaling pathways	Ran↑(Barrès et al., 2010) Cdc 42↑(Zubor et al., 2020) Rac↑(Zubor et al., 2020)	ERK ? RhoB↑ (Srougi and Burridge, 2011) AKT↑(Liu et al., 2023) CXCR4↑→SDF1a↑ (Zhang et al., 2007)	Ca2+↑(Li et al., 2020a) TRPV4↑(Li et al., 2020a) LIMK↑→cofilin↑(Maekawa et al., 1999) MLC↑ (Yoneda et al., 2005) B3GNT3↑ (Tang et al., 2019)

Notwithstanding these significant strides, substantial knowledge gaps remain in the exploration of the ECT2 and RhoA/ROCK pathways and their intricate crosstalk mechanisms within gynecological tumors. The precise identification of upstream regulators and downstream effectors, as well as the molecular intricacies of their interactions, remain incompletely characterized. Notably, the role and underlying mechanisms of ECT2 in endometrial cancer represent a critical area of uncertainty, necessitating focused and in-depth investigation.

Looking forward, the genes and molecules within these signaling pathways hold substantial promise as potential therapeutic targets and prognostic biomarkers for gynecological malignancies. Future research endeavors should prioritize the development of innovative targeted therapies that precisely modulate the ECT2 and RhoA/ROCK pathways. Integration of these targeted interventions with emerging immunotherapeutic strategies offers a compelling opportunity to revolutionize the management of gynecological cancers, potentially enhancing early detection rates, improving treatment efficacy, and ultimately improving patient outcomes. Addressing the current research limitations through collaborative, interdisciplinary efforts will be instrumental in translating these findings into clinical practice and advancing the field of gynecological oncology.
